# Baseline Predictors of Early Ventricular Recovery During Hospitalization in Children with Dilated Cardiomyopathy: A Short-Term Retrospective Study

**DOI:** 10.3390/pediatric18050118

**Published:** 2026-09-08

**Authors:** Adelina-Mihaela Sorescu, Cristina Isabel Viorica Ghiță, Smaranda Stoleru, Gabriela Duică, Alin Marcel Nicolescu, Eliza Elena Cinteză, Ion Fulga, Oana Andreia Coman

**Affiliations:** 1 Department I—Functional Sciences, Pharmacology, Clinical Pharmacology and Pharmacotherapy, “Carol Davila” University of Medicine and Pharmacy, 050474 Bucharest, Romania; adelina-mihaela.enache@drd.umfcd.ro (A.-M.S.); smaranda.stoleru@umfcd.ro (S.S.); ion.fulga@umfcd.ro (I.F.); oana.coman@umfcd.ro (O.A.C.); 2 Pediatrics Department, “Carol Davila” University of Medicine and Pharmacy, 050474 Bucharest, Romania; gabrieladuica@yahoo.ro (G.D.); elizacinteza@yahoo.com (E.E.C.); 3 “Marie S. Curie” Clinical Emergency Hospital for Children, 077120 Bucharest, Romania; nicolescu_a@yahoo.com

**Keywords:** dilated cardiomyopathy, predictors, pediatric

## Abstract

Introduction: Determining the prognosis and probable evolution of dilated cardiomyopathy (DCM) in pediatric patients has been an area of interest in the literature over the years. The aim of this study was to investigate baseline clinical, laboratory, and echocardiographic parameters associated with early ventricular recovery during hospitalization in children with dilated cardiomyopathy. Materials and methods: A retrospective, observational, single-center study was conducted including 56 pediatric patients diagnosed with DCM between 2015 and 2025. Baseline clinical and paraclinical variables were collected at admission. Changes in the left ventricular ejection fraction were assessed during hospitalization, and patients were categorized into recovery and non-recovery groups. Statistical analysis was performed to identify independent predictors of early ventricular recovery. Results: Early ventricular recovery occurred in 25 (44.64%) patients. In multivariate logistic analysis, Ross functional class was the only independent marker associated with early ventricular recovery (OR 2.547, 95% CI 1.179–5.501; *p* = 0.017). Bootstrap validation confirmed the robustness of the association. Troponin, NT-proBNP, mitral regurgitation and treatment strategy were not independently associated with recovery. ROC analysis demonstrated a modest discriminatory performance for the Ross functional class. Discussion: In this cohort of children with DCM, symptom severity was the only baseline variable consistently associated with early ventricular recovery. Clinical assessment was more useful than laboratory biomarkers and echocardiographic parameters in predicting short-term improvement in ventricular function. Conclusions: The Ross functional class was the only baseline variable independently associated with early ventricular recovery. These findings support the use of the Ross functional classification as a simple bedside tool, useful in early prognostic stratification in children with dilated cardiomyopathy, although its discriminatory performance was modest.

## 1. Introduction

Dilated cardiomyopathy is the most common primary cardiomyopathy in children and represents an important cause of pediatric heart failure [1]. With an incidence of 0.6–0.7 cases per 100,000 children, aged 0–18 years, dilated cardiomyopathy is a rare form of heart failure in children [2,3,4].

Multiple etiologies have been implicated in the development of dilated cardiomyopathy over the years. Based on its causes, dilated cardiomyopathy has thus been classified either as primary (familial or genetic causes) or secondary (due to cardiovascular or systemic diseases such as arrhythmic causes, infections, myocarditis, metabolic diseases, nutritional causes and toxins) [5,6,7,8,9,10,11]. Despite these recognized causes, the etiology of dilated cardiomyopathy remains unclear in over 50% of the cases [12,13,14].

Regardless of its etiology, dilated cardiomyopathy is defined by two processes: ventricular chamber dilation (increase in left ventricular end-diastolic diameter over 2 standard deviations for age) and systolic dysfunction (decrease in the ejection fraction for more than 2 standard deviations for age) [5,15,16,17].

Diagnosis relies on clinical assessment, laboratory biomarkers, electrocardiography, and imaging studies, with echocardiography remaining the cornerstone of diagnosis [18,19,20,21,22,23].

Management of pediatric heart failure remains challenging because evidence-based pediatric guidelines are limited and treatment strategies are largely extrapolated from adult studies [24,25,26], in spite of important differences in disease etiology and pharmacological responses between children and adults [27,28,29].

The prognosis and clinical course of pediatric dilated cardiomyopathy have been extensively investigated over the years. Certain risk factors or negative prognostic factors have been identified. The most frequently described clinical risk factors have been: the age at diagnosis, heart rate and symptom severity (namely the Ross functional class) [3,17,30]. Aside from these, laboratory tests such as NT-proBNP and cardiac markers have been implicated in providing insight into disease progression [20,31,32,33,34,35]. The left ventricular diastolic dimension, the ventricular systolic function and the degree of mitral regurgitation are only some of the imaging parameters used to determine the prognosis of dilated cardiomyopathy [12,36,37].

In dilated cardiomyopathy, disease progression can be highly variable, with three main possible clinical courses: at least partial recovery of ventricular function, long-term clinical stability or rapid progression towards heart transplantation or death [15,38].

However, most available studies have focused on long-term outcomes such as transplantation, persistent ventricular dysfunction, or mortality, whereas factors associated with short-term ventricular recovery during hospitalization remain poorly characterized. Early identification of patients with a higher likelihood of ventricular improvement may facilitate clinical decision-making and improve risk stratification.

Therefore, the aim of this study was to investigate baseline clinical, laboratory, and echocardiographic parameters associated with early ventricular recovery during hospitalization in children with dilated cardiomyopathy. Secondary objectives were to identify possible independent predictors of recovery and to evaluate the discriminatory performance of these variables, as well as to determine the association between treatment strategy and echocardiographic improvement.

## 2. Materials and Methods

### 2.1. Study Design and Population

A single-center, retrospective, observational study was conducted in pediatric patients diagnosed with dilated cardiomyopathy and admitted to the Department of Pediatric Cardiology, “Marie S. Curie” Emergency Clinical Hospital for Children, Bucharest, Romania, between January 2015 and January 2025.

Eligible patients were aged between 0 and 18 years and fulfilled echocardiographic criteria for dilated cardiomyopathy, defined as left ventricular dilation (left ventricular end-diastolic diameter Z-score > +2) associated with left ventricular systolic dysfunction (left ventricular ejection fraction [LVEF] < 55%). Patients were included regardless of the underlying etiology of dilated cardiomyopathy.

Patients were excluded if they had perinatal hypoxia, isolated congenital heart disease, genetic syndromes, endocrine disorders, other systemic diseases, sepsis or severe systemic infection, other forms of cardiomyopathy, coronary artery abnormalities, prior cardiac surgery, ventricular assist devices, pacemakers, implantable cardioverter-defibrillators, or incomplete clinical data.

### 2.2. Data Collection

Patient identification was performed using International Classification of Diseases, Tenth Revision (ICD-10) discharge codes for cardiomegaly (I51.7), cardiomyopathies (I42, I42.0, I42.8, I42.9), and heart failure (I50.0, I50.1, I50.9). All identified cases were subsequently reviewed manually to confirm eligibility. Clinical and laboratory data were extracted retrospectively from archived medical records and discharge summaries.

The following baseline variables were collected at hospital admission: age, gender, duration of hospitalization, Ross functional class, creatine kinase (CK), creatine kinase MB fraction (CK-MB), troponin, N-terminal pro-brain natriuretic peptide (NT-proBNP), left ventricular ejection fraction (LVEF), and severity of mitral regurgitation.

### 2.3. Clinical and Echocardiographic Assessment

Heart failure severity was assessed at admission using the Ross classification [39]. Patients were categorized into four functional classes, ranging from class I (asymptomatic) to class IV (symptoms at rest), with higher classes indicating more severe heart failure.

Reference values for serum CK, CK-MB, troponin and NT-proBNP levels were provided by the institution’s laboratory. CK levels were considered high when above 270 U/L and CK-MB levels, above 25 U/L. A troponin level above 40 ng/mL was considered elevated, while a high NT-proBNP level was above 125 pg/mL.

Echocardiographic examinations were performed by a certified pediatric cardiologist according to institutional practice and European and American guidelines for echocardiographic assessment [24,25]. Left ventricular systolic function was assessed by measuring LVEF using the Simpson biplane method. Systolic dysfunction was defined as an LVEF <55%.

Mitral regurgitation severity was classified according to vena contracta width as mild (<3 mm), moderate (3–7 mm), or severe (>7 mm).

### 2.4. Treatment Strategy

Medical treatment was prescribed according to the attending physician and institutional protocols. All patients received loop diuretics (Furosemide, at a starting dose of 1 mg/kg/day) during hospitalization. For descriptive and exploratory analyses, patients were categorized into two treatment groups: patients receiving ACE inhibitors and aldosterone receptor blockers during hospitalization were considered treatment group A, while patients receiving ACE inhibitors, beta-blockers and aldosterone receptor blockers were considered treatment group B.

### 2.5. Definitions

The primary outcome of the study was early ventricular recovery during hospitalization.

Early ventricular recovery was defined as an increase in left ventricular ejection fraction of at least 10 percentage points between hospital admission and discharge, according to the institutional protocol. This threshold was selected to identify clinically meaningful improvement in left ventricular systolic function while minimizing the influence of interobserver variability and normal measurement fluctuations associated with echocardiographic assessment. A ≥10-percentage-point increase in LVEF has also been used as a criterion for ventricular functional improvement or recovery in previous studies of patients with dilated cardiomyopathy [40].

Patients were subsequently categorized into a recovery group and a non-recovery group according to this definition.

### 2.6. Ethical Considerations

The study was conducted in accordance with the principles of the Declaration of Helsinki. Ethical approval was obtained from the Ethics Committee of the “Marie S. Curie” Emergency Clinical Hospital for Children, Bucharest, Romania (approval number 18329/31 March 2026). In accordance with institutional and local regulations, written informed consent was obtained at admission from the parents or legal guardians of all participants, regarding admission, clinical and paraclinical assessment, treatment and the use of anonymized clinical data for educational and scientific purposes.

### 2.7. Statistical Analysis

Continuous variables were tested for normality and expressed as mean ± standard deviation or median with interquartile range (IQR), as appropriate according to data distribution. Categorical variables were presented as frequencies and percentages. Continuous variables were assessed for normality using the Shapiro–Wilk test.

Baseline characteristics were compared between patients with and without early ventricular recovery. Continuous variables were analyzed using the Mann–Whitney U test, while categorical variables were compared using Fisher’s exact test, according to data distribution.

Age, Ross functional class, troponin, NT-proBNP, and mitral regurgitation severity were selected based on clinical relevance and previously reported associations with outcomes in pediatric dilated cardiomyopathy and were entered into multivariable logistic regression models to identify independent predictors of early ventricular recovery. Given the limited sample size, the number of variables included in multivariable models was restricted to avoid model overfitting. Bootstrap validation was used to confirm the robustness of the analyzed models. Results were reported as odds ratios (ORs) with 95% confidence intervals (CIs).

Receiver operating characteristic (ROC) curve analysis was performed to evaluate the discriminatory performance of baseline variables for predicting early ventricular recovery. The area under the curve (AUC), 95% confidence intervals, sensitivity, and specificity were calculated.

Changes in left ventricular ejection fraction during hospitalization were assessed by comparing admission and discharge values.

A two-sided *p*-value < 0.05 was considered statistically significant. Statistical analyses were performed using SPSS version 20 (IBM Corp., Armonk, NY, USA).

## 3. Results

### 3.1. Baseline Characteristics of the Study Population

A total of 56 children diagnosed with dilated cardiomyopathy were included in the study. The baseline demographic, clinical, biological and echocardiographic characteristics of the study population are summarized in Table 1.

The mean age at admission was 6.8 years and 33 patients (58.9%) were male. Most patients presented with symptomatic heart failure (49 patients, 87.5%), with 13 patients (23.2%) classified as Ross functional class II, 17 patients (30.35%) classified as functional class III and 19 patients (33.92%) were classified as Ross functional class IV.

Echocardiographic assessment demonstrated impaired left ventricular systolic function, with a median baseline LVEF of 25% (IQR 17.5, 32.5). Mitral regurgitation was assessed at admission in each patient and 51 patients (91.1%) had some degree of mitral regurgitation.

Baseline laboratory evaluation revealed elevated cardiac biomarker levels with median CK, CK-MB, troponin and NT-proBNP concentrations of 101 U/L, 27.15 U/L, 40 ng/mL and 11,918 pg/mL, respectively.

Regarding treatment strategy, 24 patients (42.85%) received treatment with ACE inhibitors and aldosterone receptor blockers, while 32 patients (57.15%) received ACE inhibitors, aldosterone receptor blockers and beta-blockers.

### 3.2. Early Improvement in Left Ventricular Systolic Function

**Figure 1:**
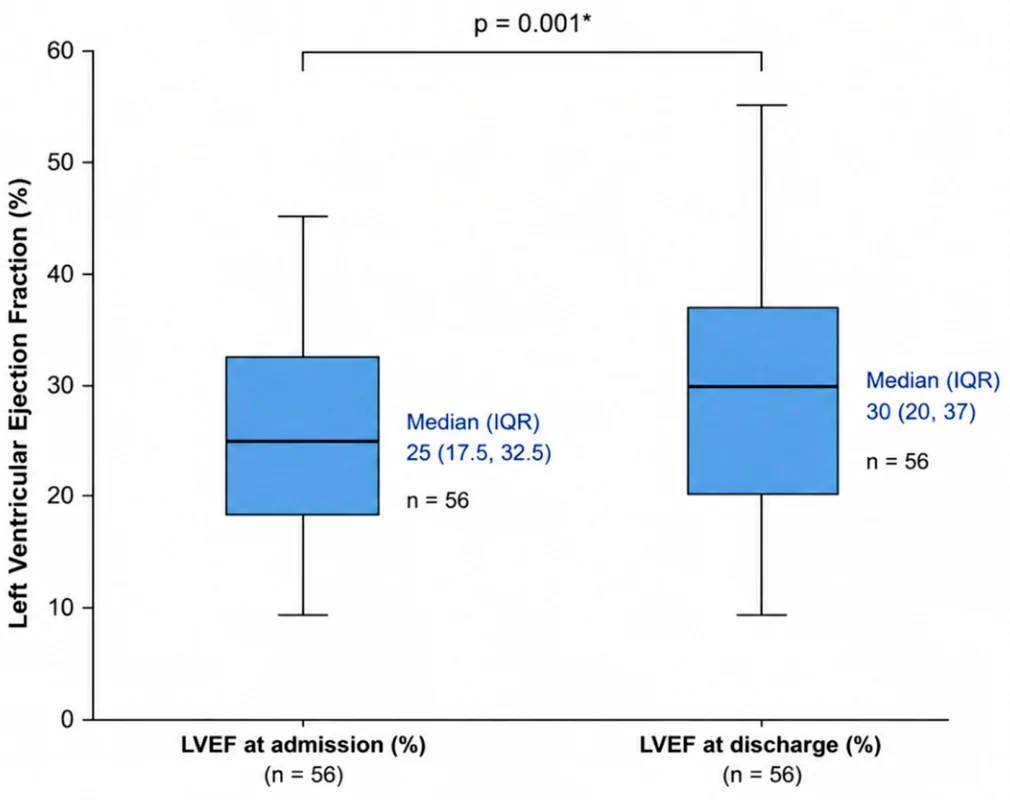
Change in left ventricular ejection fraction from admission to discharge; LVEF, left ventricular ejection fraction. Statistically significant values are marked by “*”.

A significant improvement in left ventricular systolic function was observed during hospitalization. Median LVEF increased from 25% (IQR 17.5, 32.5) at admission to 30% (IQR 20, 37) at discharge (*p* < 0.05) (Table 2) (Figure 1).

The overall distribution of LVEF values shifted significantly upward during hospitalization, 25 patients (44.64%) achieved early ventricular recovery, whereas 31 patients (55.36%) did not demonstrate significant improvement in systolic function.

### 3.3. Baseline Factors Associated with Early Ventricular Recovery

Baseline clinical, biological, echocardiographic and treatment-related characteristics were compared between patients with and without early ventricular recovery, as shown in Table 3.

Patients who achieved early systolic function improvement had a significantly different distribution of Ross functional classes at admission, with a higher proportion of patients in advanced Ross classes. No statistically significant differences were observed between the two groups regarding age, gender, cardiac biomarker levels, baseline mitral regurgitation or treatment strategy (Table 3).

### 3.4. Predictors of Early Ventricular Recovery

Univariate logistic regression analyses were performed to evaluate the association between baseline variables and early ventricular recovery. Ross functional class was significantly associated with the likelihood of recovery (OR 2.026, 95% CI 1.063–3.862; *p* = 0.032), whereas troponin, NT-proBNP, mitral regurgitation and treatment strategy did not demonstrate statistically significant associations. Variables with clinical relevance were subsequently included into multivariable models (Table 4).

In the first multivariable model (Model 1), which included Ross functional class, troponin and NT-proBNP levels and mitral regurgitation severity, the Ross functional class emerged as the only independent predictor of early ventricular recovery (OR 2.547, 95% CI 1.179–5.501; *p* = 0.017). Troponin showed a non-significant trend toward association (*p* = 0.089), whereas age, NT-proBNP and mitral regurgitation were not independently associated with recovery. The overall model was statistically significant (Omnibus test *p* = 0.05) and demonstrated good calibration (Hosmer–Lemeshow *p* = 0.766) (Table 4).

To assess the robustness of the multivariable model given the limited sample size, bootstrap resampling (1000 iterations) was performed. Ross functional class remained significantly associated with early ventricular recovery (bootstrap OR 2.26, 95% CI 1.22–7.66; *p* = 0.018), supporting the stability of the observed association.

Additional adjustment for treatment strategy (Model 2) did not improve model performance and did not identify treatment as an independent predictor of early ventricular recovery. Given the limited sample size and number of outcome events, the loss of statistical significance in the treatment-adjusted model is thus likely related to reduced model stability (Table 4).

### 3.5. Diagnostic Performance of Baseline Biomarkers

Receiver operating characteristic (ROC) curve analysis was performed to assess the discriminatory ability of baseline variables for identifying patients who achieved early ventricular recovery (Table 5). Ross functional class demonstrated modest discriminatory ability with an AUC of 0.665 (95% CI 0.521–0.808; *p* = 0.036). A cutoff value corresponding to Ross class III or higher provided the optimal balance between sensitivity (88%) and specificity (54.8%). Troponin, NT-proBNP levels, mitral regurgitation severity and treatment strategy showed poor discriminatory ability, with AUC values close to 0.50 and non-significant results.

### 3.6. Association Between Treatment Strategy and Early Ventricular Recovery

The proportion of patients achieving early ventricular recovery did not differ significantly among treatment groups (Table 6). In treatment group A (ACE inhibitors and aldosterone receptor blockers), 12 patients (50%) had improved systolic function at discharge, while 12 patients (50%) did not. In treatment group B (ACE inhibitors, aldosterone receptor blockers and beta-blockers) 13 patients (40.62%) had improved systolic function, while 19 patients (59.37%) did not. No significant association was observed between treatment strategy and the likelihood of ventricular recovery (*p* = 0.59).

These findings were consistent with the univariate and multivariable logistic regression analyses, where adjustment for treatment strategy did not improve model performance and treatment was not independently associated with the likelihood of early ventricular recovery.

## 4. Discussion

Dilated cardiomyopathy remains one of the leading causes of heart failure in children and is characterized by a highly heterogeneous clinical course, ranging from complete recovery to progressive heart failure requiring transplantation or resulting in death. Identifying early prognostic markers is therefore essential for risk stratification and clinical decision-making [5,6,27].

In this retrospective cohort of children with dilated cardiomyopathy, early ventricular improvement during hospitalization was observed in approximately 44.64% of patients. The main finding of our study was that Ross functional class at admission emerged as the only independent predictor of early ventricular recovery. This association remained consistent across multivariable models and after adjustment for treatment strategy. In contrast, baseline troponin, NT-proBNP, mitral regurgitation severity, age, and treatment strategy were not independently associated with recovery.

Studies evaluating pediatric heart failure treatment are limited. Consequently, pediatric heart failure management is largely based on evidence derived from adult populations [1,28].

The evolution and prognosis of dilated cardiomyopathy in children are highly variable, and often unpredictable [15]. Clinical as well as paraclinical risk factors have been involved in determining the evolution of dilated cardiomyopathy [12,20,32,33,37].

Symptom severity has long been recognized as an important prognostic marker in pediatric heart failure. The Ross classification remains the most widely used clinical tool for assessing heart failure severity in children and has been incorporated into routine clinical practice. Previous studies have similarly identified symptom severity at presentation as an important determinant of outcome in pediatric heart failure. Our findings extend these observations by suggesting that Ross functional class may also be useful for predicting short-term ventricular recovery during hospitalization. Notably, Ross functional class was the only variable that remained significant in all multivariable models and was also the only parameter demonstrating significant discriminatory ability in ROC analysis. This consistency across comparative analyses, multivariable regression models, and ROC analysis strengthens the robustness of the observed association.

In our cohort, higher Ross functional class was independently associated with a greater likelihood of early ventricular recovery. Although this observation may appear counterintuitive, it may reflect the greater potential for measurable improvement among patients presenting with more severe ventricular dysfunction and heart failure symptoms at admission. Conversely, patients with milder symptoms may have had less opportunity for substantial improvement in LVEF during the relatively short hospitalization period. Furthermore, bootstrap validation yielded similar effect estimates and confirmed the statistical significance of Ross functional class, supporting the robustness of this finding despite the relatively small sample size.

Several laboratory biomarkers have been proposed as predictors of outcome in pediatric dilated cardiomyopathy [41]. Studies concerning long-term prognosis have identified elevated troponin and natriuretic peptide levels as markers of adverse outcomes, including persistent ventricular dysfunction, transplantation, and death in pediatric dilated cardiomyopathy [20,31,42].

In this study, however, in a short-term setting, neither troponin nor NT-proBNP remained independently associated with early ventricular recovery after adjustment for other clinical variables. Although troponin demonstrated a non-significant trend toward association, the overall discriminatory performance of these biomarkers was limited. These findings suggest that clinical assessment at presentation may provide more prognostic information regarding short-term ventricular recovery than isolated biomarker measurements.

In addition to biological investigations, echocardiographic parameters play an important role in determining the diagnosis, treatment and evolution of dilated cardiomyopathy. In particular, systolic dysfunction and the degree of left ventricular dilation have been frequently reported as prognostic factors in pediatric dilated cardiomyopathy [12,35,36].

Although mitral regurgitation is frequently regarded as a marker of disease severity, it was not independently associated with early ventricular recovery and it did not retain independent prognostic significance after adjustment for clinical variables, suggesting that symptom burden at presentation may better reflect the likelihood of short-term recovery than isolated echocardiographic findings [43].

ROC curve analysis demonstrated modest discriminatory ability for Ross functional class, with an AUC of 0.665. In contrast, troponin, NT-proBNP, mitral regurgitation severity, and treatment strategy showed poor predictive performance. Although the discriminatory capacity of Ross functional class was only moderate, its ease of assessment and independent association with recovery support its potential role as a modest practical bedside prognostic marker. Nevertheless, the AUC value below 0.70 suggests that Ross functional class should be viewed as a supportive prognostic tool rather than a standalone predictor.

Several studies from the Pediatric Cardiomyopathy Registry have identified clinical and echocardiographic variables associated with long-term outcomes, including transplantation, persistent ventricular dysfunction, and mortality. In particular, Molina et al. demonstrated that baseline ventricular function and heart failure severity were associated with disease progression [33], while Everitt et al. reported that recovery of ventricular function may occur in a subset of children with idiopathic dilated cardiomyopathy during long-term follow-up [15]. In contrast to these registry studies, which primarily evaluated long-term clinical outcomes, the present study focused specifically on early ventricular recovery during hospitalization. Despite these methodological differences, our findings support the importance of baseline clinical assessment, as the Ross functional class emerged as the only variable independently associated with short-term ventricular recovery.

In a 2017 analysis of patients enrolled in the Pediatric Cardiomyopathy Registry, Rusconi et al. compared outcomes across different etiologies of dilated cardiomyopathy. The authors found no differences between patients with familial and idiopathic dilated cardiomyopathy [44]. In our study, due to the heterogeneity of the etiologies, we were unable to determine whether early ventricular improvement rates differ among etiologies.

The primary objective of the present study was not to compare treatment efficacy, but to explore baseline predictors of early ventricular recovery. Regarding treatment strategy, no significant association with early ventricular recovery was observed. Furthermore, adjustment for treatment strategy did not improve the performance of the multivariable models or materially alter the association between Ross functional class and recovery. These findings should be interpreted with caution, as treatment allocation was not randomized and may have been influenced by baseline disease severity and physician preference. Therefore, the absence of a statistically significant association should not be interpreted as evidence of equivalent treatment efficacy.

From a clinical perspective, our findings suggest that a simple bedside assessment using the Ross functional classification may provide useful prognostic information regarding short-term ventricular recovery. Given its ease of application and widespread use in pediatric practice, Ross classification may represent a practical tool for early risk stratification in children hospitalized with dilated cardiomyopathy.

These findings should, however, be regarded as hypothesis-generating and require validation in larger multicenter prospective studies.

## 5. Limitations

This study has several limitations. First, its retrospective single-center design may have introduced selection and information bias. Second, the relatively small sample size reflects the rarity of pediatric dilated cardiomyopathy and may have limited the statistical power to detect weaker associations. The relatively small number of outcome events limited the complexity of multivariable modeling and may have increased the risk of model overfitting. Third, treatment allocation was not randomized and may have been influenced by baseline disease severity and physician preference. As a result, the observed lack of association between treatment strategy and early ventricular recovery should be interpreted with caution. The etiology of dilated cardiomyopathy was heterogeneous and included patients with different underlying causes, which may have distinct clinical courses and recovery patterns. Additionally, the underlying condition was not determined in all patients included in the study, as genetic testing was not available in all patients due to technical issues at the time of diagnosis. Because of the relatively small sample size and the large number of etiological subgroups, etiology was not included in the multivariable models to avoid model instability and overfitting. Therefore, the potential influence of etiology on early ventricular recovery cannot be excluded and should be further investigated in larger multicenter studies. Finally, the study focused on short-term ventricular recovery during hospitalization and did not evaluate long-term outcomes such as persistent ventricular dysfunction, heart transplantation, or mortality.

Despite these limitations, the study provides additional data regarding potential prognostic markers of early ventricular recovery in pediatric dilated cardiomyopathy. Larger multicenter prospective studies are needed to validate the prognostic role of Ross functional class and to further evaluate clinical, laboratory, and echocardiographic predictors of recovery in this population.

## 6. Conclusions

In children hospitalized with dilated cardiomyopathy, Ross functional class was the only independent predictor of early ventricular recovery and demonstrated modest discriminatory performance in ROC analysis. Baseline biomarkers, mitral regurgitation severity, age, and treatment strategy were not independently associated with recovery. These findings support the use of clinical evaluation as a simple bedside tool for early prognostic stratification in children with dilated cardiomyopathy, although its discriminatory performance was modest. Larger multicenter prospective studies are needed to validate the prognostic role of clinical, laboratory, and echocardiographic predictors of recovery in pediatric dilated cardiomyopathy.

## Figures and Tables

**Table 1:** Baseline demographic, clinical, biological and echocardiographic characteristics of the study population.

Characteristics of the Patients Studied (*n* = 56)	
**Variable**	
**Demographics**	
Female, *n* (%)	23 (41.1)
Male, *n* (%)	33 (58.9)
Age, years	6.8 ± 0.88
**Clinical**	
Hospitalization duration, days	17.66 ± 2.97
Symptomatology at admission, *n* (%)	49 (87.5)
Ross Class, *n* (%)	
Class I	7 (12.5)
Class II	13 (23.2)
Class III	17 (30.35)
Class IV	19 (33.92)
**Echocardiography**	
Baseline LVEF, median (Q1, Q3)	25 (17.5, 32.5)
Mitral regurgitation *n* (%)	
No mitral regurgitation	5 (8.9)
Mild	8 (14.3)
Moderate	23 (41.1)
Severe	20 (35.7)
**Paraclinical**	
CK, median (Q1, Q3)	101 (71.75, 154)
CK-MB, median (Q1, Q3)	27.15 (20.25, 38.1)
Troponin, median (Q1, Q3)	40 (40, 75)
NT-proBNP, median (Q1, Q3)	11,918 (1805.5, 29,185)
**Treatment strategy**	
Group A, *n* (%)	24 (42.85)
Group B, *n* (%)	32 (57.15)

Continuous variables are reported as mean ± standard deviation or median with interquartile range, according to data distribution. Abbreviations: CK, creatine kinase, CK-MB, creatine kinase MB, NT-proBNP, N-terminal pro-B-type natriuretic peptide, LVEF, left ventricular ejection fraction.

**Table 2:** Changes in left ventricular systolic function during hospitalization.

Parameter	Admission	Discharge	*p*-Value
LVEF (%)	25 (IQR 17.5, 32.5)	30 (IQR 20, 37)	0.001 *

Data are presented as median with interquartile range, according to data distribution. Abbreviations: LVEF, left ventricular ejection fraction. Statistically significant values are marked by “*”.

**Table 3:** Baseline characteristics according to early ventricular recovery status.

Variable	Recovery LVEF	Non-Recovery LVEF	*p*-Value
Age, years (mean)	7.2 ± 1.2	6.5 ± 1.28	0.65
Male (*n*)	13	20	0.34
Ross class (*n*)			
Class I	1	2	0.04 *
Class II	2	12	
Class III	10	8	
Class IV	12	9	
CK (median)	97 (72, 125.75)	106.5 (69.75, 188.2)	0.54
CK-MB (median)	26.4 (19.45, 40.40)	27.5 (21.75, 39)	0.94
Troponin (median)	40 (40, 74.25)	40 (40, 81)	0.64
NT-proBNP (median)	12,321 (2673, 22,279)	11,560 (1455, 32,535)	0.63
Mitral regurgitation (*n*)			
No MR	2	3	0.54
Mild MR	3	4	
Moderate MR	10	13	
Severe MR	9	11	
Treatment (*n*)			
Group A	12	12	0.59
Group B	13	19	

Data are presented as mean ± standard deviation, median (IQR) or number as appropriate. Between group comparisons were performed using the Mann–Whitney U test for continuous variables and Fisher’s exact test for nominal variables. Statistically significant values are marked by “*”. Abbreviations: CK, creatine kinase; CK-MB, creatine kinase myocardial band; NT-proBNP, N-terminal pro-B-type natriuretic peptide; LVEF, left ventricular ejection fraction; MR, mitral regurgitation.

**Table 4:** Univariate and multivariable logistic regression analyses of predictors of early ventricular recovery.

Variable	Univariate OR (95% CI)	*p*	Model 1 Adjusted OR (95% CI)	*p*	Model 2 Adjusted OR (95% CI)	*p*
Age	1 (0.994–1.007)	0.932	-	-	-	-
Ross class	2.026 (1.063–3.862)	0.032 *	2.547 (1.179–5.501)	0.017 *	2.543 (1.178–5.490)	0.017 *
Troponin	1.002 (0.998–1.006)	0.346	1.004 (0.99–1.009)	0.089	1.004 (0.99–1.009)	0.123
NT-proBNP	1 (1–1)	0.815	1 (1–1)	0.186	1 (1–1)	0.201
MR	1.009 (0.571–1.784)	0.975	0.678 (0.347–1.323)	0.255	0.679 (0.348–1.324)	0.256
Treatment	0.827 (0.485–1.410)	0.486	-	-	0.923 (0.507–1.681)	0.793

Odds ratios (ORs) and 95% confidence intervals (CIs) are presented. Model 1 included Ross functional class, troponin, mitral regurgitation severity, and NT-proBNP. Model 2 was additionally adjusted for treatment strategy. Bootstrap estimates were obtained using 1000 samples. Statistically significant values are marked by “*”. Abbreviations: OR, odds ratio; CI, confidence interval; NT-proBNP, N-terminal pro-B-type natriuretic peptide; MR, mitral regurgitation.

**Table 5:** Receiver operating characteristic analysis of baseline variables for prediction of early ventricular recovery.

Variable	AUC (95% CI)	*p*-Value
Ross class	0.665 (0.521–0.808)	0.036 *
Troponin levels	0.543 (0.381–0.688)	0.662
NT-proBNP levels	0.463 (0.309–0.616)	0.633
Mitral regurgitation	0.500 (0.347–0.653)	0.99
Treatment strategy	0.454 (0.301–0.607)	0.553

ROC curve analysis of baseline variables for prediction of early ventricular recovery. The statistically significant results are marked in bold. Statistically significant values are marked by “*”. Abbreviations: AUC, area under the curve; CI, confidence interval; NT-proBNP, N-terminal pro-B-type natriuretic peptide.

**Table 6:** Association between treatment strategy and early ventricular recovery.

Treatment Group	Early Improvement	No Improvement
Group A	12 (50%)	12 (50%)
Group B	13 (40.62%)	19 (59.37%)

Data are presented as number of patients (%). Associations between treatment strategy and early ventricular recovery were assessed using Fisher’s exact test.

## Data Availability

The data presented in this study are available upon request from the corresponding author.
